# Cyclosporine in the treatment of childhood idiopathic steroid resistant nephrotic syndrome: a single centre experience in Nigeria

**DOI:** 10.11604/pamj.2016.25.258.9802

**Published:** 2016-12-29

**Authors:** Taiwo Augustina Ladapo, Christopher Imokhuede Esezobor, Foluso Ebunoluwa Lesi

**Affiliations:** 1 Department of Pediatrics, College of Medicine, University of Lagos, PMB 12003, Idi-Araba, Lagos, Nigeria; 2 Lagos University Teaching Hospital, PMB 12003, Idi-Araba, Lagos, Nigeria

**Keywords:** Nephrotic syndrome, steroid resistant, idiopathic, childhood, immunosuppresives, cyclosporine

## Abstract

**Introduction:**

Children with steroid resistant nephrotic syndrome usually require treatment with second-line agents and calcineurin inhibitors such as cyclosporine are now recommended as initial therapy. These agents only recently become available in our environment and their impact on care is unknown. We reviewed the short-term treatment outcomes of their use in comparison with previous outcomes.

**Methods:**

Medical records of children managed for idiopathic steroid resistant nephrotic syndrome over a 5 year period were reviewed. Remission rates and improvement in renal function following use of various agents were compared.

**Results:**

Of 103 children with idiopathic nephrotic syndrome, 25(24.3%) were steroid resistant, of whom 17 received additional medications. Full remission rate for cyclosporine was 70% (7/10). Remission rates prior to the availability of cyclosporine were 40% (2/5) for cyclophosphamide and 66% (2/3), (partial remission only) with enalapril, an angiotensin converting enzyme inhibitor used in combination with alternate day prednisolone. One child with cyclophosphamide resistance subsequently achieved remission with cyclosporine. Remission was not related to sex *(p=0.96)*, age *(p=0.54)*, serum albumin *(p=0.37)* or hypertension *(p=0.43)* but to serum cholesterol *(p= 0.02)*. The estimated glomerular filteration rate (eGFR) among children treated with cyclosporine ranged from 30-167 ml/min/1.73m^2^ as follows: >90 (5); 60-89 (3); 30-59 (2) while the mean pre and post treatment eGFR in those with eGFR <90 were 60 and 104ml/min/1.73m^2^ respectively *(p=0.03)*. Mortality rate was 10% (1/10) in children treated with cyclosporine compared with 28.6% (2/7) in those treated with other medications *(p=0.54)*.

**Conclusion:**

Cyclosporine resulted in improved treatment outcomes in children with idiopathic steroid resistant nephrotic syndrome.

## Introduction

Nephrotic Syndrome (NS) is a glomerular disease characterized by severe proteinuria, hypoalbuminemia, anasarca and hypercholesterolemia [1]. The idiopathic form accounts for about 90% of cases and responds to first-line therapy with corticosteroids in about 80% of affected children [1]. The remaining steroid resistant group often constitutes a management challenge with higher incidences of infections, complications of steroid toxicity as well as progression to Chronic Kidney Disease (CKD) and End Stage Kidney Disease (ESKD) [2].

Efforts to improve the outcome of children with idiopathic steroid resistant nephrotic syndrome (iSRNS) have resulted in the emergence of various steroid sparing agents with considerations for sustained remission and reduced side effects. These include alkylating agents such as cyclophosphamide (CYC), calcineurin inhibitors such as cyclosporine (CsA) and tacrolimus, mycophenolate mofetil (MMF), a T and B cell proliferation inhibitor and rituximab, a monoclonal antibody generally reserved for multi-therapy resistant cases. These have been used singly or in combination with wide variations in practice among pediatric nephrologists worldwide and consequently, varying outcomes [3–6]. In order to standardize care, the Kidney Disease: Improving Global Outcomes (KDIGO) group [7] in 2012, made several recommendations on the treatment of childhood idiopathic SRNS. These include the use of calcineurin inhibitors as the first line drugs for the management of steroid resistant nephrotic syndrome which we have since adopted at our centre. The objective of this study therefore was to determine the short-term outcome of the treatment of childhood iSRNS with cyclosporine, and compare this with outcome prior to adoption of the KDIGO guidelines.

## Methods

### Study centre

The study was conducted at the Paediatric Nephrology Unit of the Lagos University Teaching Hospital in Southwest Nigeria. Data of all children diagnosed with sephrotic syndrome (serum albumin<25g/L, spot urine protein: creatinine ratio >200mg/mmol, generalized oedema and serum cholesterol > 5.2mmol/l) [1, 7] between January 2009 and December 2014 was analyzed. Children who were followed up for less than 3 months or those with incomplete data were excluded from the study. Ethical approval was obtained from the health research and ethics committees of the hospital and the study conformed to the ethical guidelines and principles of the Helsinki Declaration of 2008.

#### Patient management protocol

At first presentation, demographic data, history and clinical findings of all patients were documented. Beside confirmatory tests for nephrotic syndrome as earlier stated, other tests routinely done to assess disease severity, complications and diagnose possible aetiologies include serum electrolytes, urea, creatinine, calcium, phosphate, urinalysis and urine microscopy, culture and sensitivity, hemoglobin genotype, screening for hepatitis B, C and Human Immunodeficiency Virus and renal ultrasound scan. Chest radiographs were done to detect latent tuberculosis which may be exacerbated by steroids. Antibody to double stranded DNA, C3, C4 and ANCA were only performed, due to cost constraints, as indicated. Glomerular filtration rate (eGFR) was estimated in using the modified Schwartz formula [8]. Chronic kidney disease (CKD) was defined according to the Kidney Disease Outcomes Qualitative Initiative (KDOQI) guidelines [9]. A diagnosis of idiopathic nephrotic syndrome was made in the absence of a secondary cause for nephrotic syndrome.

#### Treatment regimen

All patients with idiopathic NS received oral prednisolone at 60mg/m2 daily for 4-6 weeks. Since 2012, following the KDIGO recommendations,(7) we have extended treatment to 8 weeks to define steroid resistance. Response to treatment was defined as follows: ((1) ***remission:*** proteinuria <30mg/dl (trace or nil) for 3 consecutive days; ***steroid resistant nephrotic syndrome*** (SRNS): no remission after 6 weeks of daily prednisolone, and after 8 weeks since 2012; ***relapse***: recurrence of 100mg/dl (≥2+) proteinuria for 3 consecutive days after initial remission; ***steroid dependent nephrotic syndrome*** (SDNS): two consecutive relapses during alternate day steroid therapy or within 14 days after cessation of steroids; ***Frequently relapsing nephrotic syndrome*** (FRNS): two or more relapses within 6 months of initial response or ≥4 relapses in any 12-month period. Following remission the dose is reduced to 40mg/m^2^ on alternate days for 4 weeks and gradually tapered over 3-5 months. In steroid resistant cases, a kidney biopsy was performed in some cases before treatment with one of the following treatment regimens: (1) Enalapril, at a starting dose of 100mcg/kg/day. (2) Intravenous cyclophosphamide, 500mg/m2/monthly for 6 months, or oral cyclophosphamide, 2 mg/kg/day for 8 week. (3) Cyclosporine at a starting dose of 5.0 mg/kg/day and titrated according to response and serum drug levels. (Our first option since 2012). All medications were given in combination with low dose alternate day prednisolone.

CsA, serum levels were checked two to four weeks after commencing the medication and then subsequently 2-3 monthly or as indicated as cost permitted. Dosages were adjusted to maintain trough levels at 70-120ng/ml. CsA resistance was diagnosed following failure of remission after 6 months of treatment. Primary outcome of treatment with CsA was remission at 6months while secondary outcome was progression to end-stage kidney disease.

#### Statistical analysis

Data were analyzed using the Statistical Package for Social Sciences software version 20. Continuous data were represented as means and standard deviations or median and range as appropriate while categorical data were presented as percentages. Chi-square test was used to determine the association between categorical data while student t test was used for comparison of means. Correlation between some variables and likelihood of remission was determined using Pearson’s correlation co-efficient. Statistical significance was set <0.05.

## Results

Of the 129 children managed for NS during the study period, 103 had the idiopathic form (iNS) of whom 25(24.3%) were steroid resistant. Children with iSRNS were aged between 0.6-15.2years (median 8.8) with the majority (64%) being >5years. A summary of their baseline characteristics is shown in Table 1. Median age was significantly higher in children with idiopathic SRNS compared with the steroid sensitive form *(9.1 vs 5years respectively, p=0.008)* Two children were referred to other centers on request, one died from complications of acute kidney injury before treatment for SRNS could be commenced while five defaulted from follow-up.

**Table 1:** Demographics of children with idiopathic steroid resistant nephrotic syndrome

Parameter	No (%)
**Sex**	
Male	12(48)
Female	13(52)
**Age group(years)**	
0-5	9(36)
6-10	7(28)
>10	9(36)
Hypertension	14(56)
Serum creatinine >88 µmol/l	6 (24)
**Parameter**	**Mean(SD)/median (range)**
Median age(years)	8.8 (0.8-16)
Mean serum albumin (g/dl)	1.98±1.1
Mean serum cholesterol (mmol/l)	13.2±4.4

### Treatment of iSRNS

**Figure 1:**
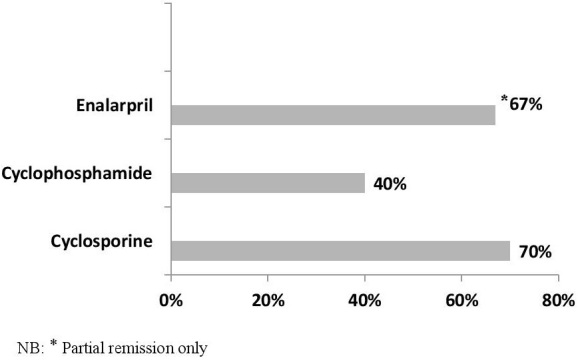
Remission rates following treatment of 17 children with idiopathic steroid resistant nephrotic syndrome

A summary of the treatment response of the remaining 17 children is shown in Table 2 and Table 3. Duration of follow up ranged from 5 months to 6.25years. Of the 5 patients treated with oral or intravenous CYC, two (40%) achieved full remission. One of these, who relapsed about a year after completing CYC subsequently responded to prednisolone. One of the non-responders subsequently achieved remission with CsA, one died from complications of sepsis while the third child relocated overseas with his parents. Three children were treated with enalapril and prednisolone of whom two achieved partial remission while the third progressed to End-stage Kidney Disease (ESKD) within a few months. She subsequently died from complications of hemodialysis. Of the 10 treated with CsA, 7(70%) achieved full remission. One had partial remission and subsequent treatment failure due to poor compliance from unaffordability of the drugs. He eventually progressed to ESKD from which he died. One child was switched to MMF because of cosmetically unacceptable facial acne as a side effect of CsA. Other side effects of CsA were hirsutism in all our patients and gingival hyperplasia in one. Till date, CsA has been discontinued in 2 children, one after 2 years of treatment and the other after 16 months due to cost-constraints. They both remain in remission 14 and 8 months respectively after discontinuation of CsA. Two children developed 1+ proteinuria at serum CsA levels <30ng/ml which resolved once the doses were increased, A summary of response to treatment is shown in Figure 1.

**Table 2:** Summary of treatment of idiopathic steroid resistant nephrotic syndrome with cyclosporine

Serial No	Sex	Age at Onset (years)	Outcome	Time to remission (weeks)	Histology
**1**	M	0.8	Remission	8	-
**2**	αF	3.0	Remission	16	FSGS
**3**	M	1.2	Remission	3	-
**4**	*+*M	4.0	Resistant	-	FSGS
**5**	F	12	Remission	12	-
**6**	M	13	Remission	20	FSGS
**7**	M	15	Remission	8	-
**8**	F	2.2	Resistant	-	MPGN
**9**	M	16	Resistant	-	FSGS
**10**	M	1.4	Remission	4	-

α **NB:** Previously resistant to cyclophosphamide

+ Secondary resistance from non-compliance

FSGS= focal segmental glomerulosclerosis; MPGN= membrano-proliferative glomerulonephritis; CsA has been discontinued in patients 2 and 5 after 24 and 16 months of treatment respectively. Patient 7 was switched to MMF because of acne and has been referred to another centre for follow-up.

**Table 3:** Summary of treatment of idiopathic steroid resistant nephrotic syndrome with other medications

Sex	Age at Onset (y)	Treatment received	Duration (months)	Outcome	Histology
F	1.9	Enalapril	12	Partial remission	MCNS
F	3.0	Enalapril	12	Partial remission	-
F	9.0	Enalapril	6	Resistant (Died)	FSGS
M	5.0	Intravenous CYC	6	Resistant(Referred)	-
F	9.1	Intravenous CYC	6	Remission	MCNS
F	6.0	Intravenous CYC	6	Resistant (Died)	-
F	3.0	+Oral CYC	2	Resistant	FSGS
F	12	Oral CYC	2	Remission	FSGS

+ **NB :** Subsequently achieved remission with cyclosporine; CYC= cyclophosphamide; MCNS=minimal change nephrotic syndrome; FSGS= focal segmental glomerulosclerosis

With regards to renal function, median serum creatinine at presentation was 56.5µmol/l with 6 children having serum creatinine greater than 88µmol/l. The estimated Glomerular filteration rate (eGFR) (ml/min/1.73m^2^) of children treated with CsA ranged from 30-167(median-78ml/min/1/73m^3^) as follows: >90=5 children (normal); 60-89= 3 children (mild decrease); 30-59=2 children (moderate decrease). The pre and post-treatment eGFR among those with reduced eGFR at presentation in this group were 60 and 104 ml/min/1.73m2 respectively *(p=0.03)*. Ten children were biopsied with histology as follows: Focal segmental glomerulosclerosis (FSGS) -7; Minimal Change Nephrotic Syndrome (MCNS)-2; Membranoproliferative glomerulnephritis-1. Biopsy was not done in 7 children due to default/refusal, financial constraints or referral to other centres. Remission with either mode of treatment was not related to sex *(p=0.96)*, age *(p=0.54)*, serum albumin *(p=0.37)* or hypertension *(p=0.43)*. It was however positively correlated with serum cholesterol on admission *(p= 0.02)*. There were 4 deaths, giving a mortality rate of 16% among children with iSRNS and 3.8% of all children with idiopathic nephrotic syndrome. In children with iSRNS, mortality rate was 10% (1/10) in children treated with cyclosporine compared with 28.6% (2/7) in those treated with other medications. *(p=0.54)*

## Discussion

Our patients were predominantly steroid sensitive as we had earlier reported [10] with only 21.7% having iSRNS. In our cohort, 70% of children treated with a CsA-prednisolone combination achieved complete remission with a combination of CsA and alternate day prednisolone with only one patient progressing to ESKD due to non-compliance with medications. There was also significant improvement in eGFR following treatment in those with impaired renal function at presentation. In comparison, in the pre-CsA era, remission rate from cyclophosphamide and prednisolone was 40% while a combination of enalapril with alternate day prednisolone only achieved partial remission in 2 of 3 patients. This clearly improved outcome is reassuring, as coupled with predominant steroid sensitivity in our patients, portends more favourable outcomes.

Several studies also report similar good outcomes with use of CsA. In Tunisia [11], overall remission rates among 30 children with iSRNS following a CsA-prednisone combination was 80%, howbeit partial in 30% of children. Progression to ESRD was however higher in their study occurring in 9 (30%) patients for reasons which are not very clear. Similarly, in Japan [12], remission rates of 82-85% were reported among 30 children with iSRNS treated for 121 months. However, in this latter study [12], children with FSGS additionally received pulse methylprednisolone which may have contributed to the higher remission rates observed.

In Iran [13], complete and partial remission rates of 32.4% and 5.4% were reported among 37 children treated with CsA and prednisolone combination for 6 months while remission rates of 53% and 65% were reported from Saudi Arabia [14] and Brazil [15] respectively. It is important to highlight the fact that differences in treatment duration before diagnosis of CsA resistance in the afore-mentioned studies exist and may account for variability in reported CsA response rates. For instance, while response to CsA was determined after 4 months in Tunisia and Japan [11, 12], in Iran (13) and Brazil [15], diagnosis was made after 6months of failure of at least partial remission as recommended by the KDIGO and as was the case in the current study. Non-uniformity of access to drugs due to cost-constraints may be another contributing factor as experienced in the group from Saudi Arabia [14] who attributed their lower remission rates to inadequate therapy due to financial and social difficulties. Similarly, one patient in our cohort developed treatment failure from non-compliance excluding which our remission rate would have been 80%. The high remission rate we have nonetheless reported may not be unrelated to the fact that CsA is relatively new in our environment, hence giving little time for emergence of resistance, but this is purely speculative.

Outcome with CsA has been shown to be improved when combined with other immune-suppressants such as MMF [13, 16]. In Iran [13], among 23 children who were CsA resistant, complete remission was observed in 11 cases (47.82%) and partial remission in 2 cases (8.7%) after combined MMF and CsA therapy thus increasing the overall remission rate. Such improved outcome has also been reported with other immunosuppressant combinations [5]. We currently have limited experience with MMF as majority of our patients achieved remission with cyclosporine. Only two of our patients eventually received MMF, one of whom has been lost to follow-up while the other was transferred to another centre for follow-up. Tacrolimus, another calcineurin inhibitor is not commonly used at our centre because of it’s high cost. Rituximab, a monoclonal antibody which is generally reserved for refractory cases of NS [6] was shown in a recent meta-analysis to have a higher rate of complete remission and significantly improved relapse-free survival compared with other immunotherapies. Further studies are however required to determine its long term effects. Short term side effects of CsA in our cohort were minimal with only one patient switching to MMF on account of acne. Similarly, only one of our patients developed gum hypertrophy which was reported in 6 patients in Saudi Arabia [14]. The major constraint to the short-term use of CsA in our environment therefore is the cost, both of the drug and of serum level determination which precluded the monitoring of serum levels as desired. Long term use remains controversial because of it’s association with nephrotoxicity [17, 18], a side effect of major concern, although there are suggestions that long-term use in moderate doses with closely monitored levels can reduce it’s incidence [18]. CsA has also been associated with a high rate of relapse after discontinuation of the drug [17] and optimal duration of treatment remains undetermined.

Our combined remission rates using cyclophosphamide or enalapril with alternate day prednisolone of 50% is similar to that from another Nigerian study [19] that reported a cumulative remission rate of 57.12% with use of pulse intravenous cyclophosphamide and intravenous dexamethasone ± lisinopril or spironolactone. In contrast to this, amongst three children treated with cyclophosphamide in the USA [5], only one (33.3%) achieved partial remission while 2 were CYC resistant while in Iran [20], 10 out of 41 (24.4%) patients with iSRNS responded to cyclophosphamide. These studies demonstrate the superiority of CsA over CYC in the management of iSRNS both in efficacy and side effect profile as has been highlighted by the KDIGO [7]. In our cohort, CsA also resulted in improved renal survival as only 1 patient, who was non-compliant with treatment progressed to ESKD.

Histological patterns from our study were FSGS, MCNS and MPGN in order of predominance agreeing with the general pattern in other studies [3, 5, 11, 14, 19]. The KDIGO [7] recommends a kidney biopsy for all children with SRNS before instituting therapy, even though opinions on the relationship between histological type and response to treatment differ [11, 13]. A kidney biopsy nevertheless remains important as it aids in the diagnosis of secondary causes, is important in disease prognostication [7] and helps in identifying changes in disease epidemiology. It however does not influence the choice of medication in children with idiopathic steroid resistant nephrotic syndrome. Identifying factors that may predict outcome may be useful in prognostication. In this study, only serum cholesterol at presentation was significantly associated with remission. Lower serum albumin levels at presentation has also been negatively associated with remission [11] and even though this was so in our study, the association was not statistically significant.

A limitation of this study was our inability to correlate serum levels of CsA with remission though none of our patients experienced relapses or increased proteinuria with CsA serum levels above 50ng/ml. This study also looked only at short –term outcome hence we are unable to determine the rate of sustained remission though patients are still under follow-up.

## Conclusion

This preliminary study affirms the efficacy of cyclosporine and steroid therapy in inducing remission and improving renal function in children with idiopathic steroid resistant nephrotic syndrome in our environment. It highlights the need for controlled studies comparing outcome with other newer immunosuppressants like tacrolimus and mycophenolate mofetil. Larger, long-term studies are also recommended to enable determination of optimal duration of treatment.

### What is known about this topic

- The care of children with steroid resistant nephrotic syndrome remains challenging and requires second-line medications;
- Calcineurin inhibitors such as cyclosporine have been recommended as first line in the treatment of childhood idiopathic steroid resistant nephrotic syndrome and have been found to improve outcome of treatment in some populations.

### What this study adds

- The use of cyclosporine as first line in the treatment of childhood idiopathic steroid resistant nephrotic syndrome resulted in improved treatment outcomes in children in our environment;
- There is a need for long-term studies to determine optimal duration of treatment with cyclosporine as well as controlled studies comparing outcome with other newer immunosuppressants in our environment.
